# Supplementary material for: An archetypes approach to malaria intervention impact mapping: a new framework and example application
Source: Malar J. 2023 Apr 26;22:138. doi: 10.1186/s12936-023-04535-0 (PMC10131392; doi:10.1186/s12936-023-04535-0)
Supplement: Supplementary file 1 — Additional file 1. Full Cluster Maps. The plots below show maps and covariate summaries for cluster counts of three to 14. In the time series, solid colored lines represent the median across the archetype, shaded areas indicate the interquartile range, and dotted lines indicate the 95% variance interval. Solid black lines represent the climate values of the representative site for each archetype, also indicated as black crosses on the map. Doughnut plots show the relative vector abundance of the representative sites. Absent doughnuts indicate no mosquitoes in that site. [file 12936_2023_4535_MOESM1_ESM.pdf]

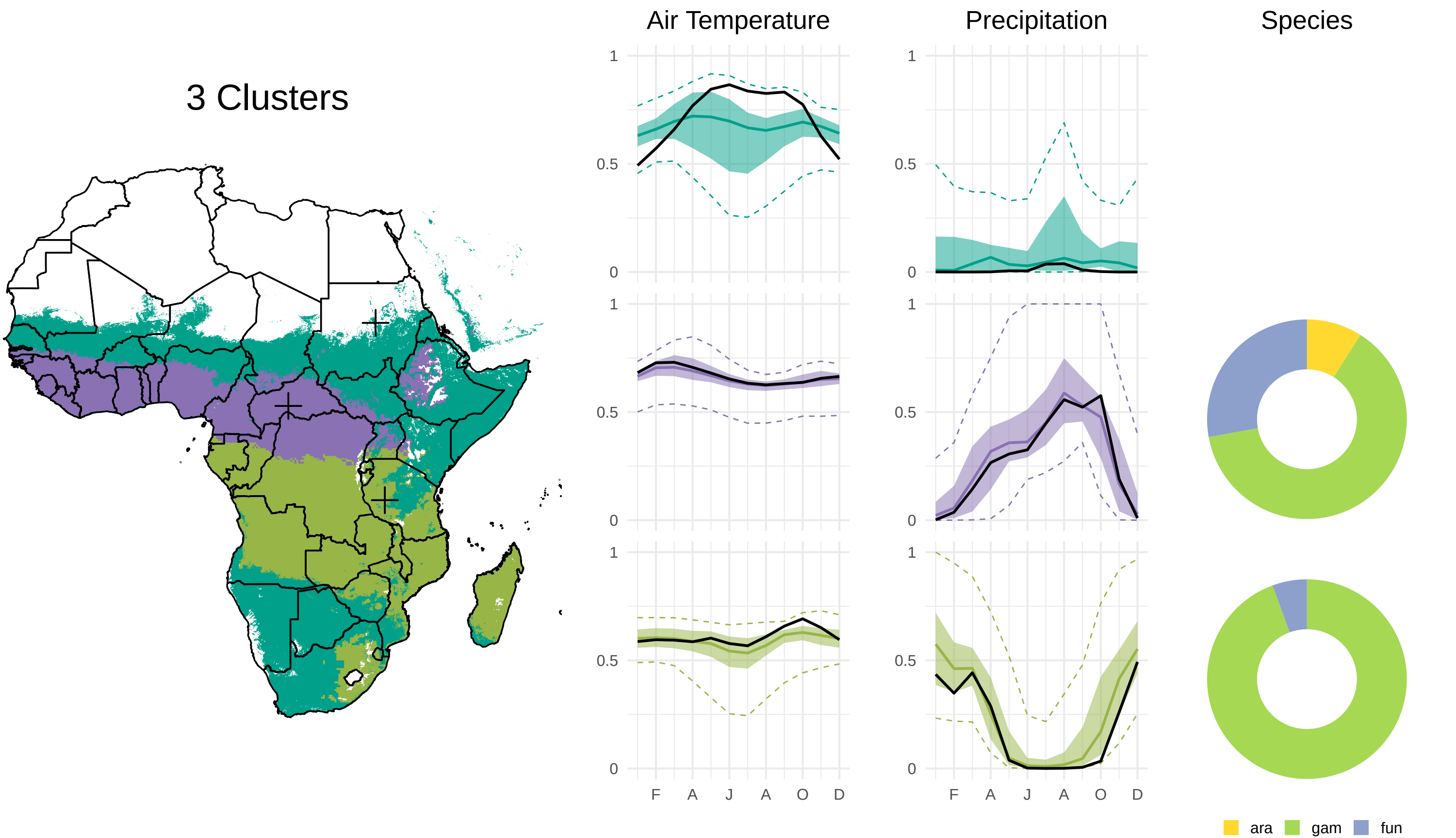
3 Clusters
Air Temperature
Precipitation
Species
1
0.5
0
F
A
J
A
O
D
ara
gam
fun

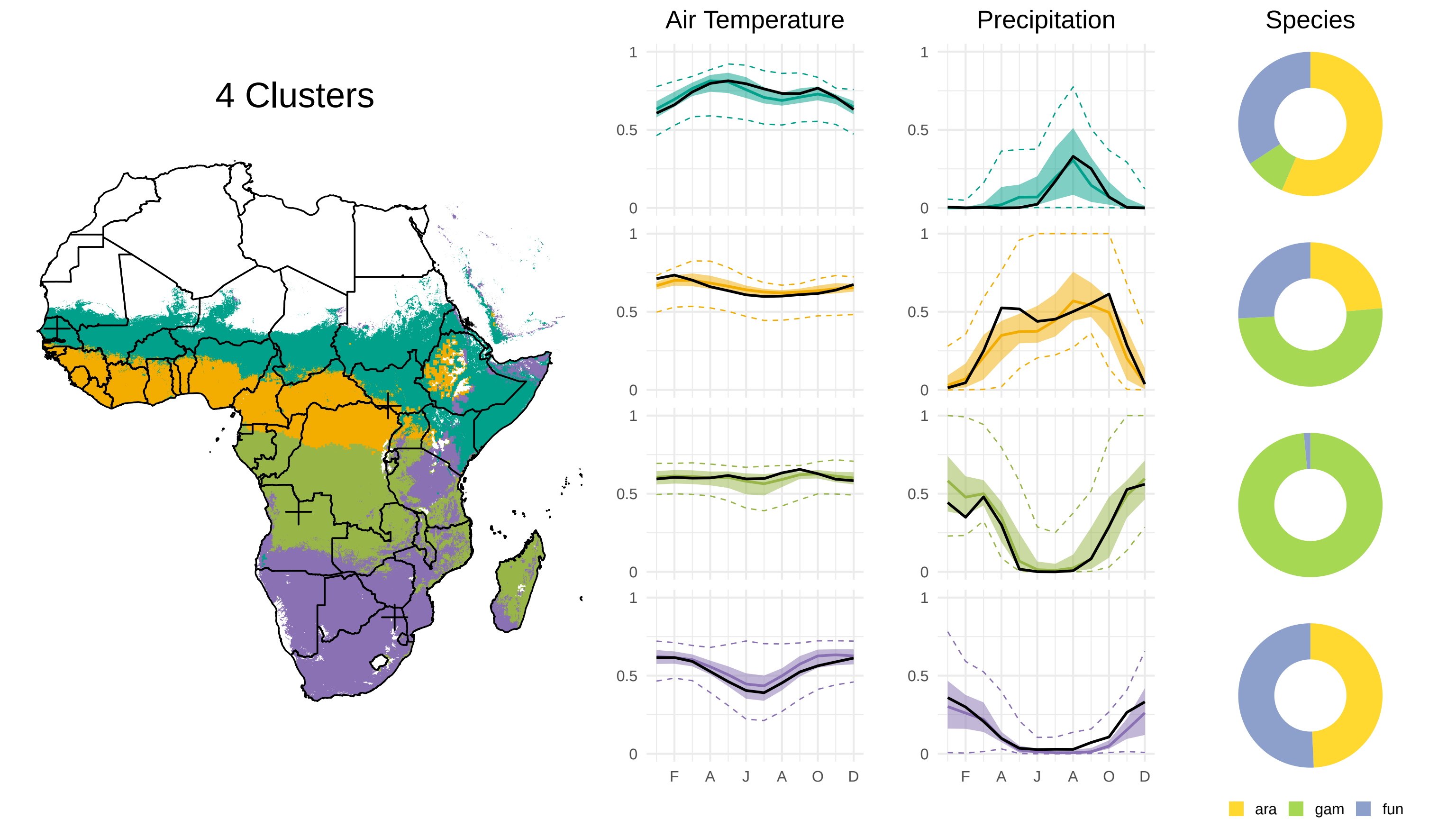
4 Clusters
Air Temperature
Precipitation
Species
1
0.5
0
F
A
J
A
O
D
ara
gam
fun

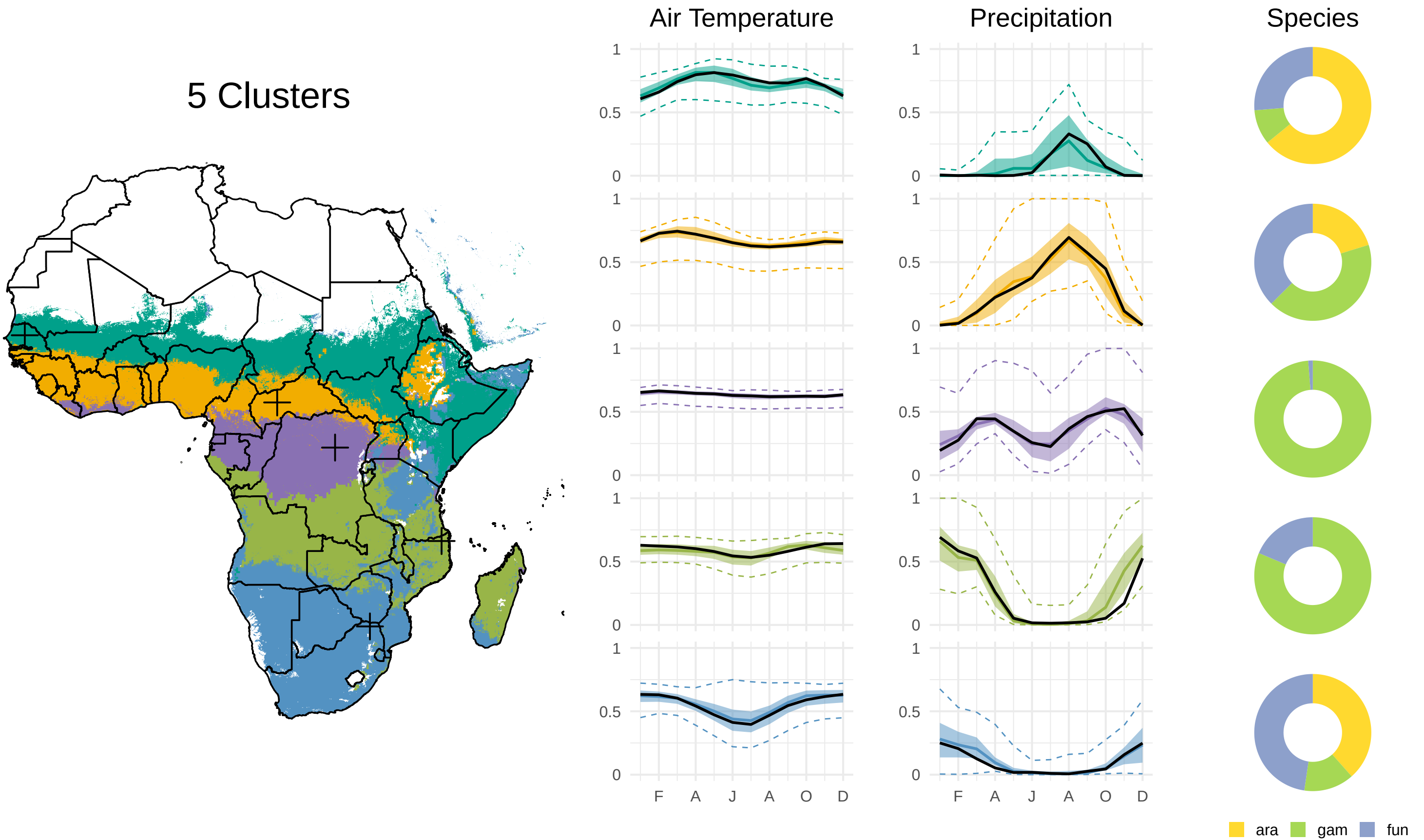
5 Clusters
Air Temperature
Precipitation
Species
1
0.5
0
F
A
J
A
O
D
ara
gam
fun

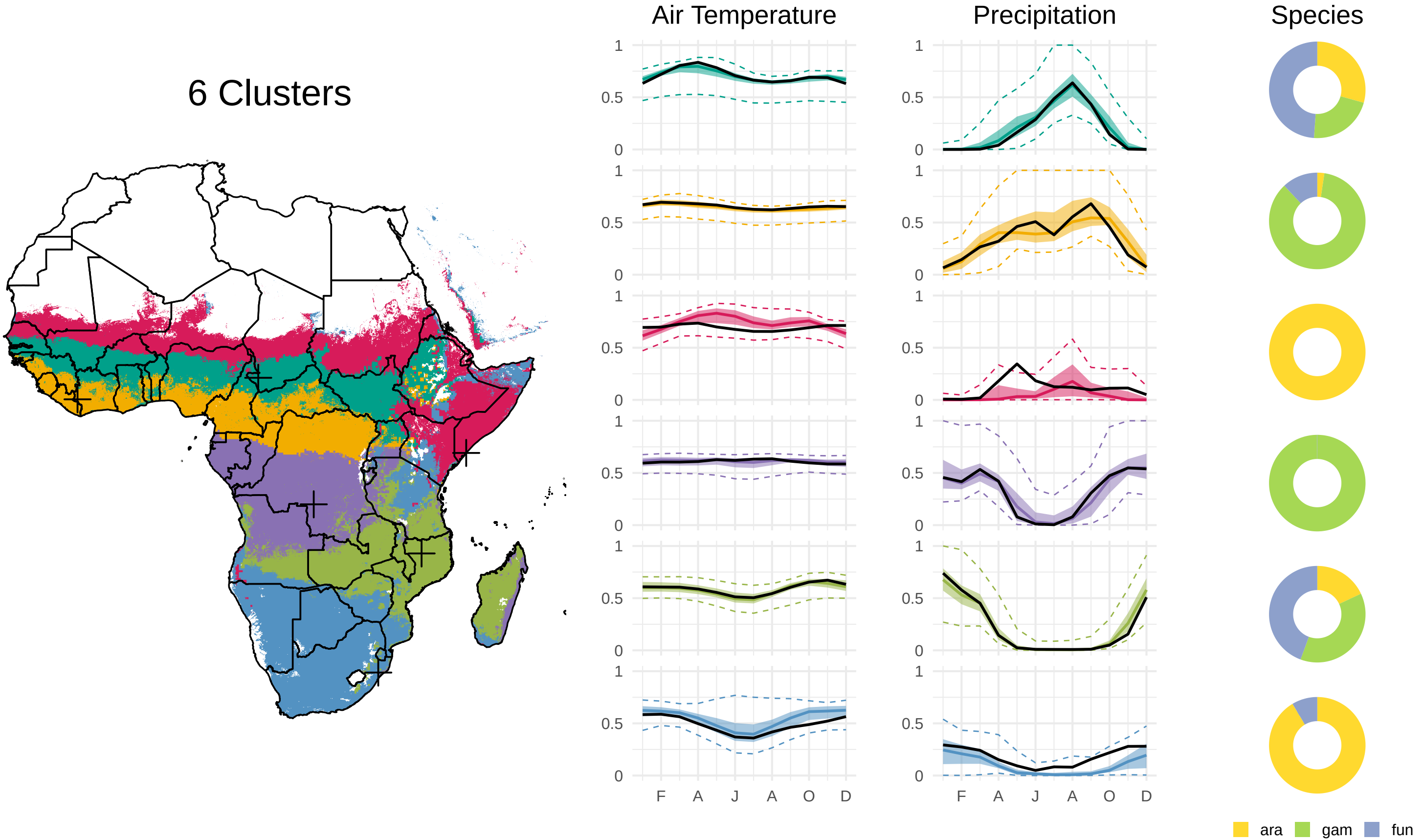
6 Clusters
Air Temperature
Precipitation
Species
1
0.5
0
F
A
J
A
O
D
ara
gam
fun

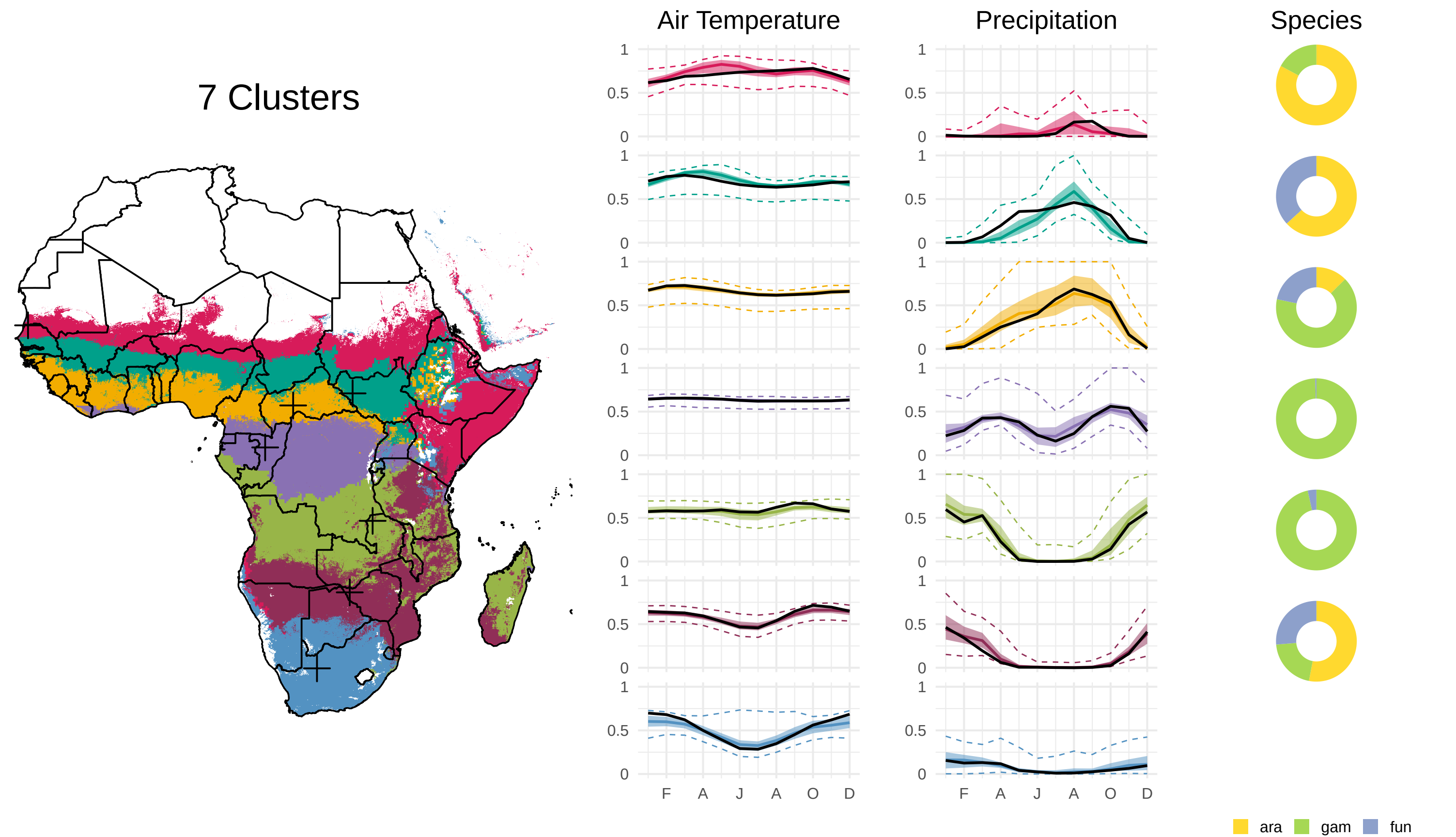
7 Clusters
Air Temperature
Precipitation
Species
1
0.5
0
F
A
J
A
O
D
ara
gam
fun

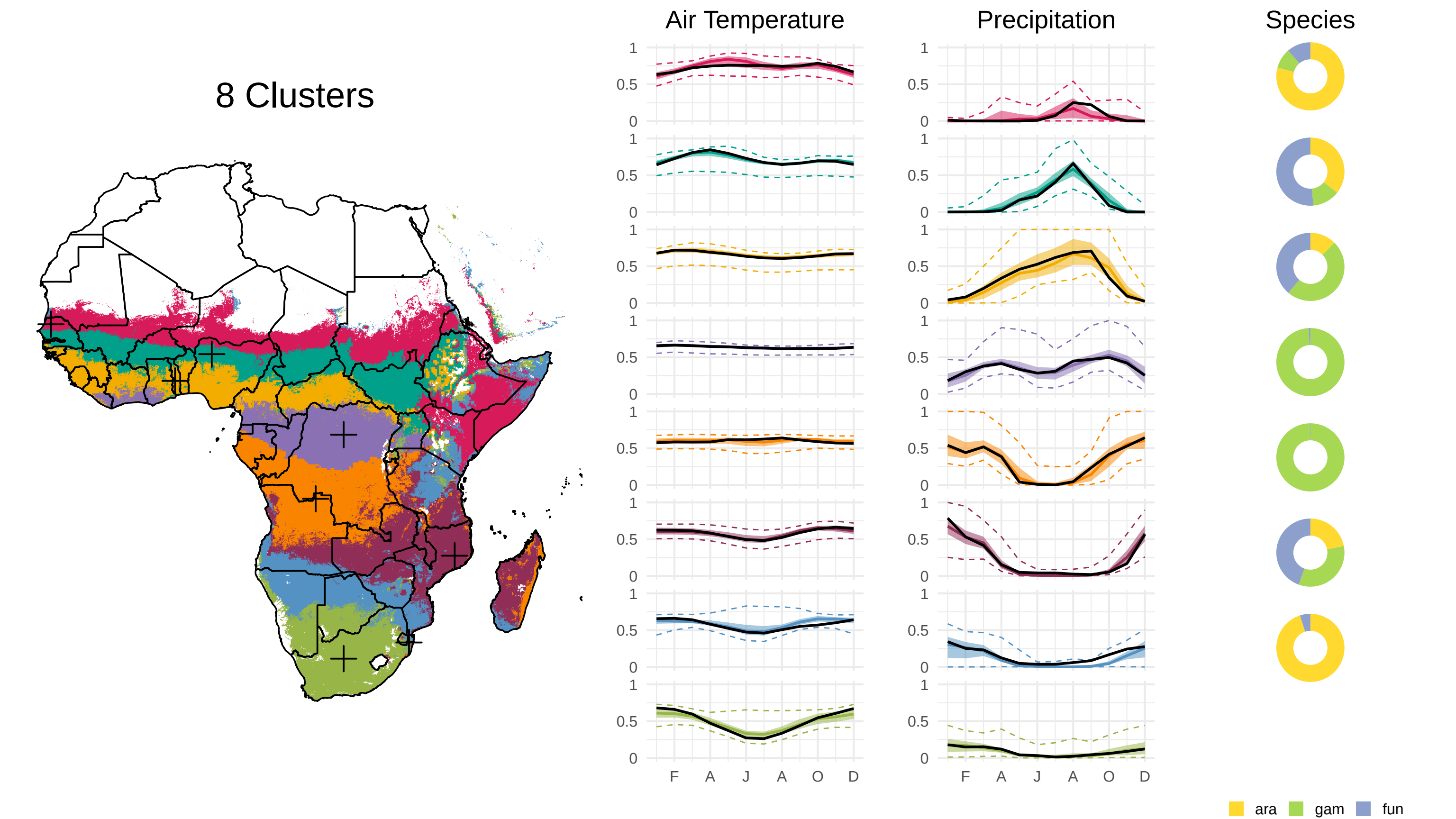

8 Clusters
Air Temperature
Precipitation
Species
1
0.5
0
F
A
J
A
O
D
ara
gam
fun

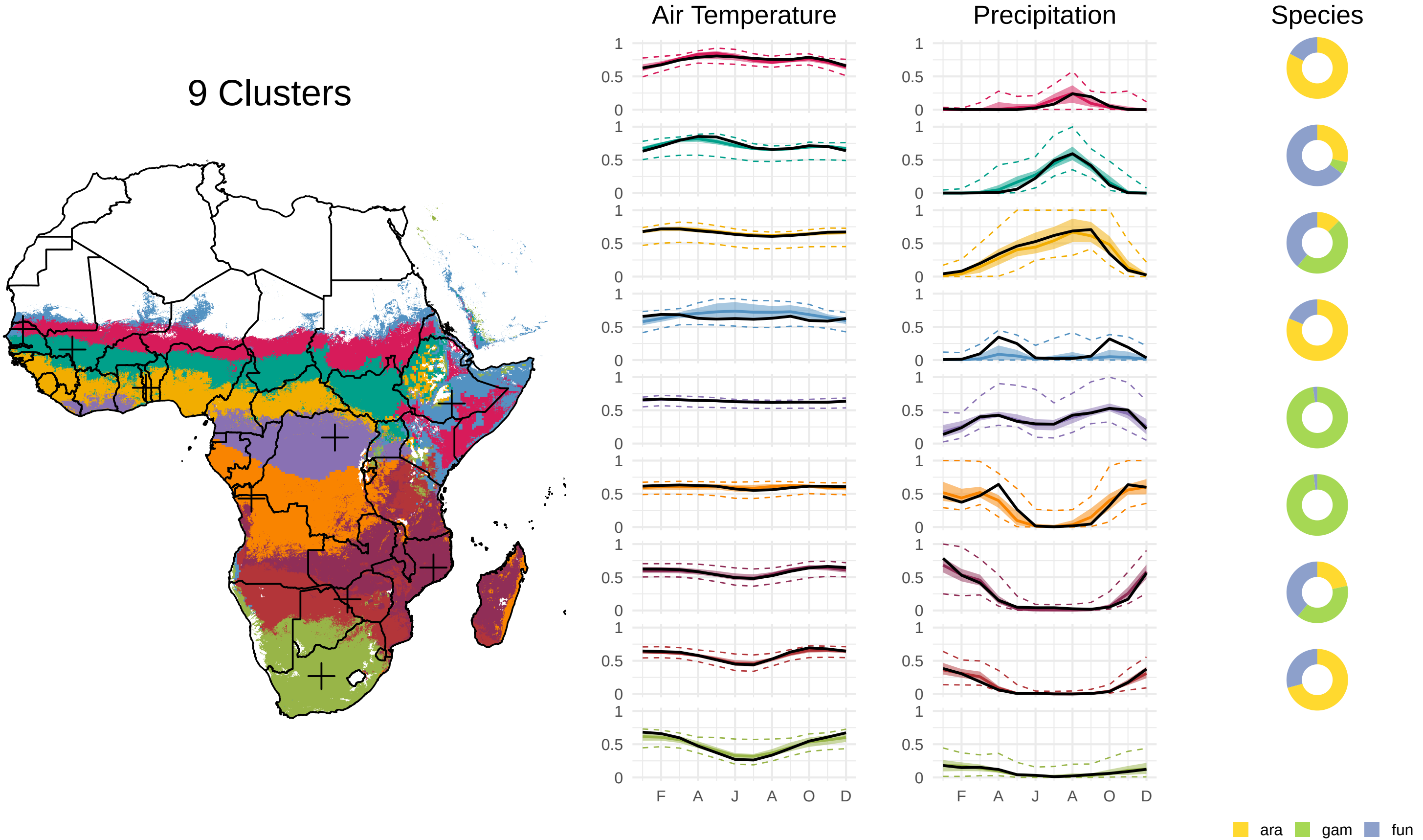
9 Clusters
Air Temperature
Precipitation
Species
F
A
J
A
O
D
ara
gam
fun

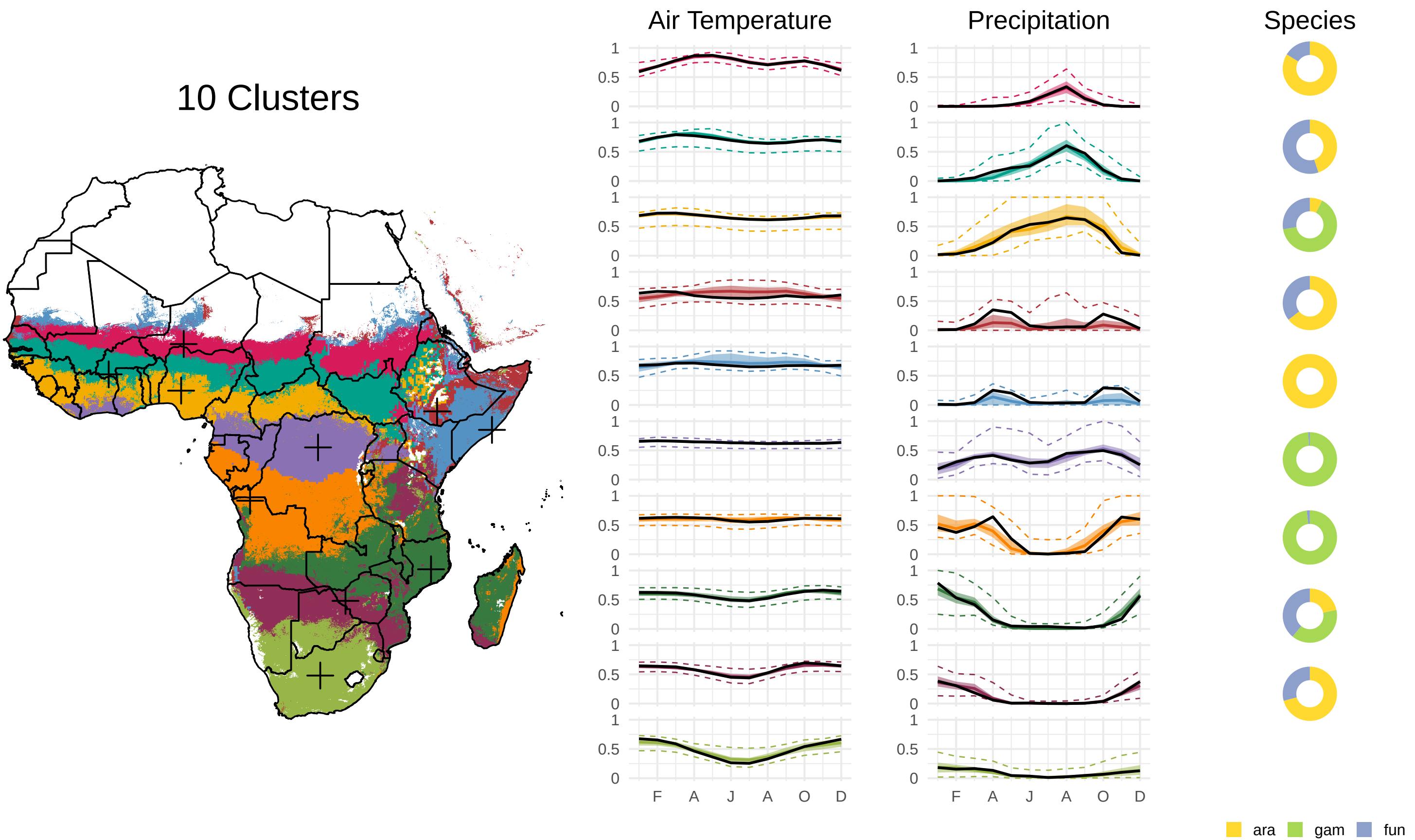
10 Clusters
Air Temperature
Precipitation
Species
1
0.5
0
F
A
J
A
O
D
ara
gam
fun

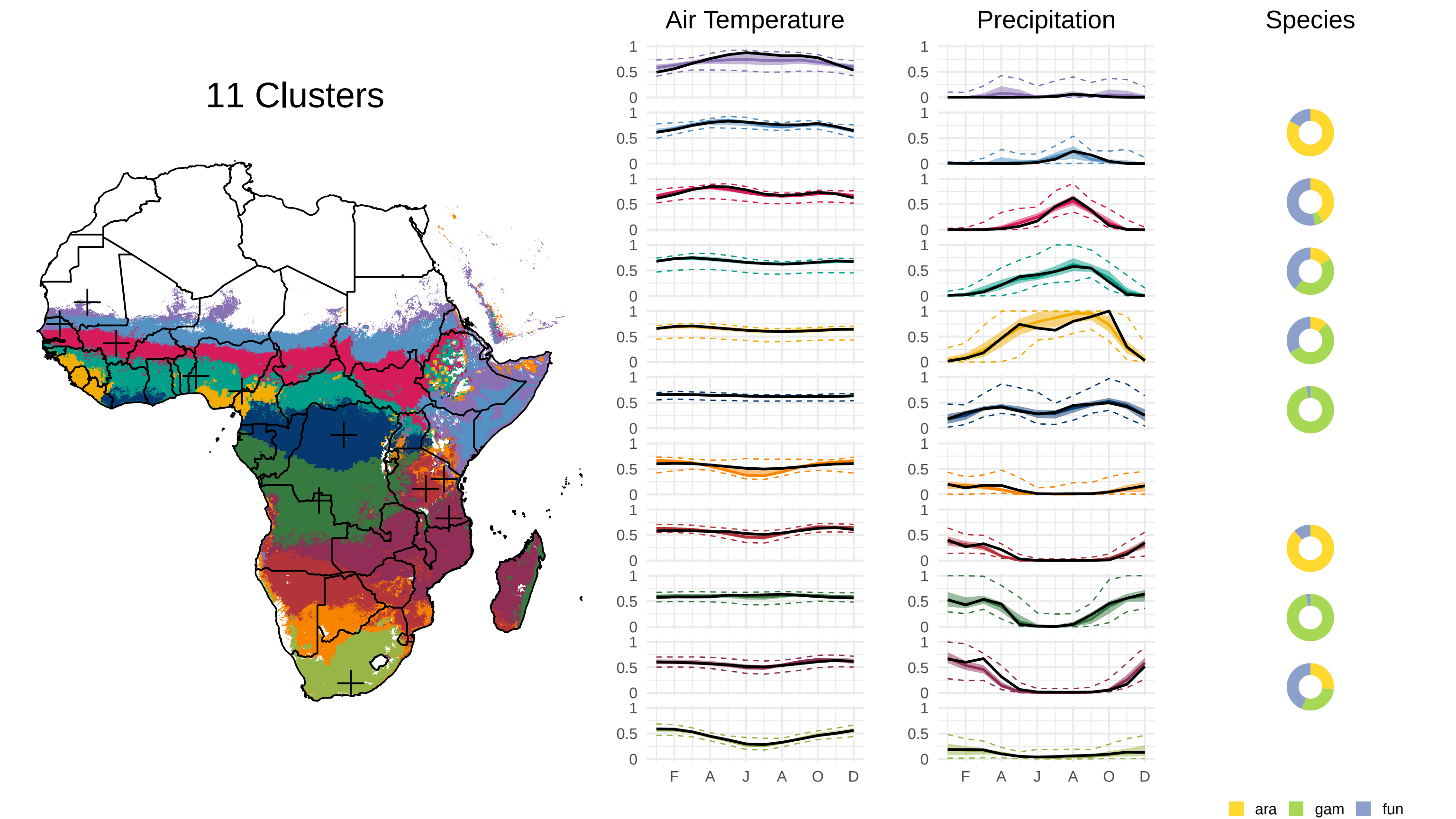

11 Clusters
Air Temperature
Precipitation
Species
1
0.5
0
F
A
J
A
O
D
ara
gam
fun

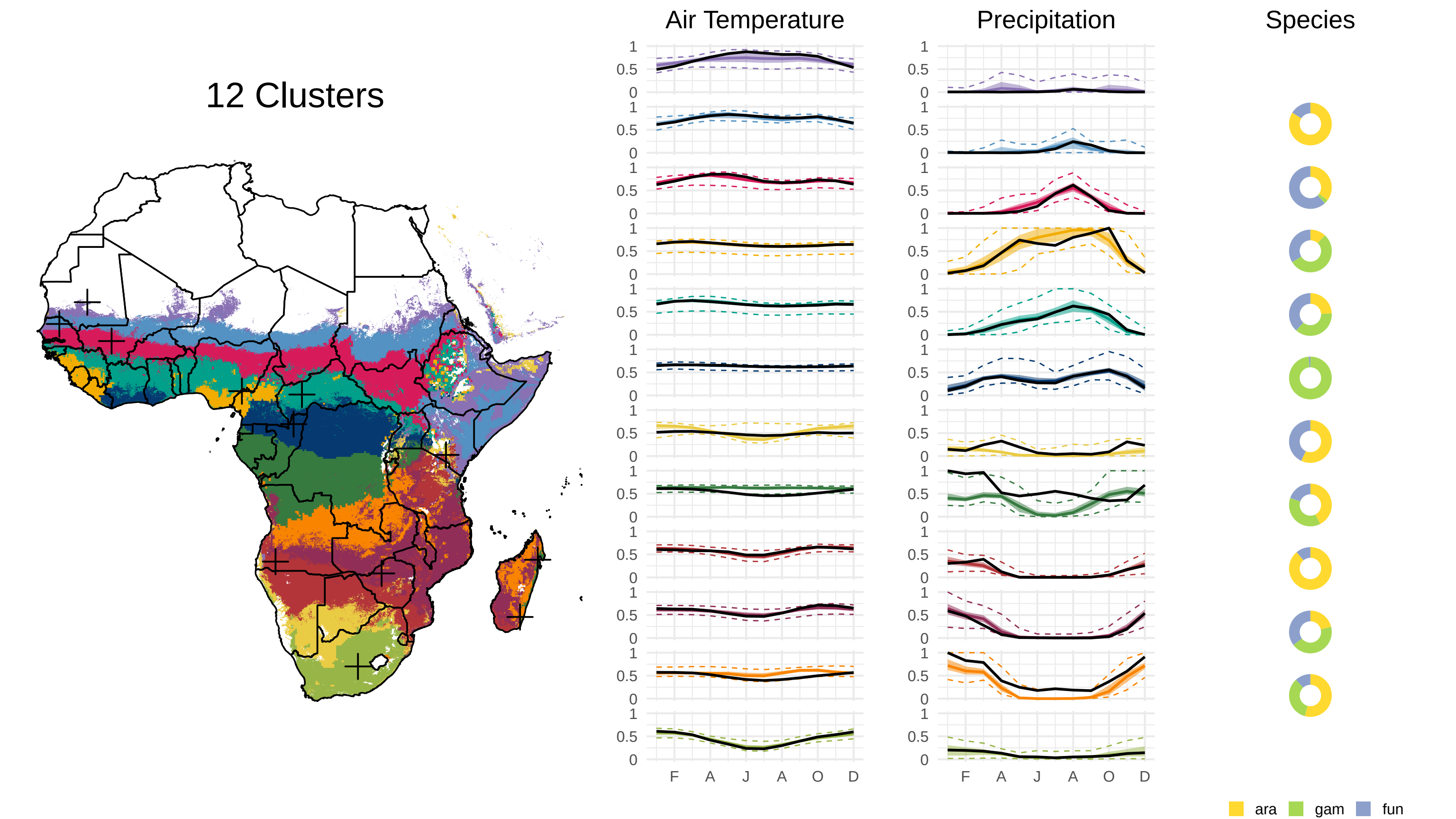

12 Clusters
Air Temperature
Precipitation
Species
1
0.5
0
F
A
J
A
O
D
ara
gam
fun

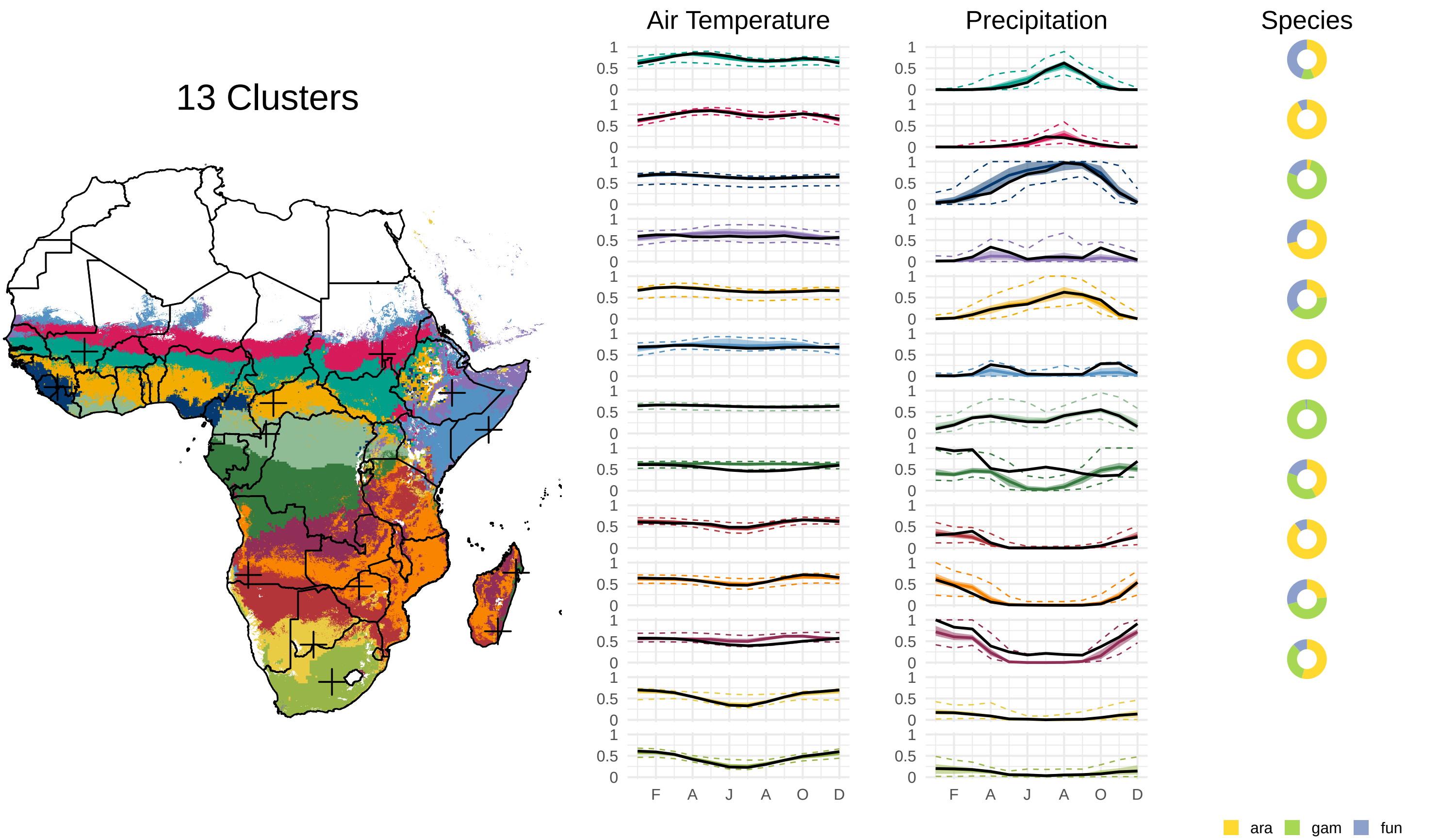
13 Clusters
Air Temperature
Precipitation
Species
F A J A O D
ara
gam
fun

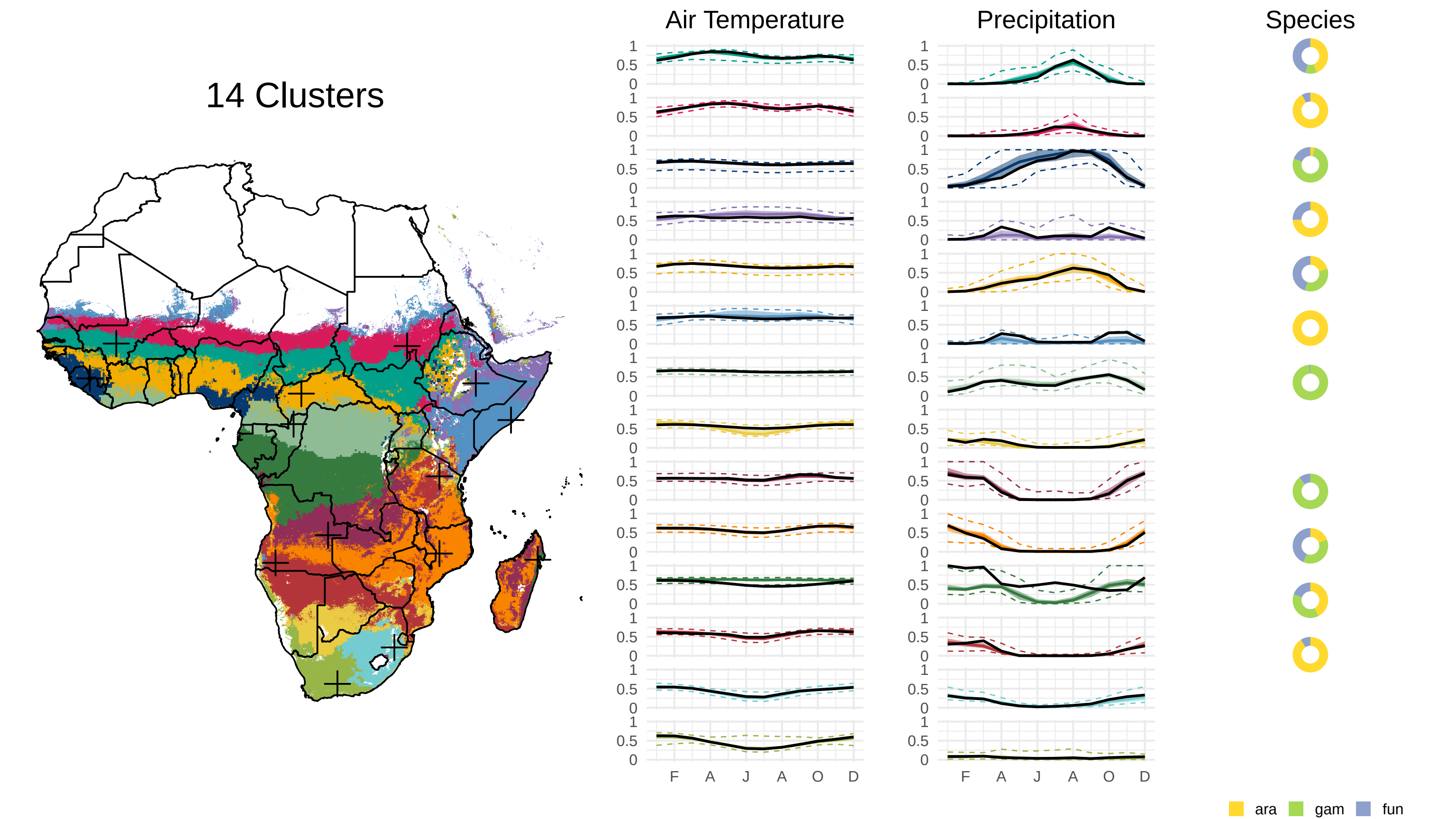
14 Clusters
Air Temperature
Precipitation
Species
1
0.5
0
F
A
J
A
O
D
ara
gam
fun
